# MKRN2 inhibits the proliferation of gastric cancer by downregulating PKM2

**DOI:** 10.18632/aging.203643

**Published:** 2022-02-23

**Authors:** Zheng Liu, Shuyao Xiang, Xingchen Guo, Jinghuan Zhou, Lixin Liao, Jiaxin Kou, Jun Zhang

**Affiliations:** 1 The Second Hospital of Lanzhou University, Lanzhou 730000, P.R. China; 2 School of Public Health, Lanzhou University, Lanzhou 730000, P.R. China; 3 Chongqing High-Tech Zone People’s Hospital, Chongqing 400000, P.R. China

**Keywords:** MKRN2, PKM2, ERK, gastric cancer, proliferation

## Abstract

Cumulative evidence suggests that dysfunction of ubiquitinating enzymes is responsible for multiple types of diseases including cancer. However, what role the ubiquitinating enzyme plays in gastric cancer remains unknown. In this study, using bioinformatics analysis and a series of experimental analyses, we found that an E3 ubiquitin-protein, MKRN2 was down-regulated in gastric cancer tissues. Kaplan–Meier survival analysis showed the low MKRN2 expression significantly indicated poor prognosis. Overexpression of MKRN2 notably inhibited cell proliferation *in vitro* and *in vivo*. Conversely, knockdown of MKRN2 had the opposite effects *in vitro*. Additionally, the mechanical analysis indicated that MKRN2 promoted ubiquitination-mediated degradation of PKM2 and attenuated its effect on ERK. Overall, the present study suggests that MKRN2 may be a potential therapeutic target for gastric cancer.

## INTRODUCTION

Gastric cancer (GC) is one of the most detrimental tumors in the digestive system, ranking fourth in morbidity and third in lethality, and >50% of GC cases occur in Eastern Asia [1–3]. However, due to the asymptomatic characteristics of the early stages of GC, diagnosis is often delayed, and the disease is diagnosed at an advanced stage, which further reduces the five-year survival rate [4, 5]. Therefore, investigating GC pathogenesis is very important to improve the clinical diagnosis and treatment of GC.

Abnormal protein degradation in cancer cells has emerged as one of the essential hallmarks of cancer and is an area of great interest in cancer research [6–8]. We found that MKRN2, an E3 ubiquitin-protein, is poorly expressed in GC from The Cancer Genome Atlas (TCGA) public database. As known, MKRN2 was involved in ubiquitin-dependent degradation of the p85α subunit of PI3K (PI3Kp85α) in lung cancer; additionally, several studies reporting that MKRN2 is a novel ubiquitin E3 ligase targeting the p65 subunit of NF-κB to inhibit the inflammatory response [9–11]. However, the association between MKRN2 expression and the development of GC remains unclear. The aim of our study is to examine the potential biological function of MKRN2 in carcinogenesis of GC.

## RESULTS

### MKRN2 is downregulated in GC and low MKRN2 levels are correlated with worse survival in patients

**Figure 1:**
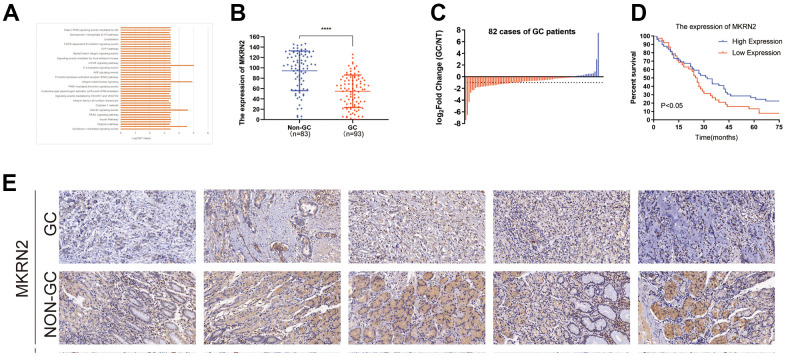
**Reduced expression of MKRN2 was found in human and gastric cancer (GC) cell line samples.** By referring to the TCGA, we found that MKRN2 expression was associated with cell signaling pathways related to cell proliferation (**A**). Expression of MKRN2 in gastric cancer tissues (n=93) as compared to adjacent tissues (n=83) (**B**, **E**). The reduced expression of MKRN2 in GC tissues as compared to adjacent issues by relative expression (the expression ratio of gastric cancer to normal tissue, GC/NT) (**C**). The expression of MKRN2 in gastric cancer tissues positively correlates with patient survival rate. Kaplan-Meier curves show patients with high MKRN2 expression have better overall survival than the patients with lower expression (**D**).

To evaluate the relationship between MKRN2 and GC, we measured the expression levels of MKRN2 in 93 resected GC tissues and 83 adjacent normal tissues via immunohistochemistry (Figure 1E). The expression of MKRN2 in GC tissues was lower than that in the adjacent tissues (Figure 1B). Among the 82 paired samples, the expression level of MKRN2 in 28 cancer tissues was only half that in the adjacent cancer tissues. In addition, the survival analysis of these patients (Figure 1D) indicated that low MKRN2 expression was associated with poor overall survival (OS) time in patients with GC.

We further evaluated the correlations between MKRN2 expression levels and clinicopathological characteristics of patients with GC. The expression of MKRN2 in patients with GC was not significantly correlated with gender, age, and TNM stage. However, downexpression of MKRN2 was found to be associated with low tumor differentiation and large tumor size, indicating that lack of MKRN2 promotes GC progression (Table 1).

**Table 1:** Association between MKRN2 and the clinicopathological characteristics of GC.

**Clinicopathological feature**	**MKRN2**		**P-value**
	**High expression**	**Low expression**	
**Gender**			
**Male**	40	30	
**Female**	10	13	0.254
**Age(years)**			
**≥60**	35	32	
**<60**	15	11	0.636
**Degree of differentiation**			
**Well, moderately**	25	11	
**Poorly, undifferentiated**	25	32	0.016
**TNM**			
**I+II**	18	16	
**III+IV**	32	27	0.904
**Tumor size (cm)**			
**≥6**	31	17	
**<6**	18	24	0.039

*In vitro* analyses revealed significantly reduced expression of MKRN2 in the four GC cell lines as compared with that in the normal gastric cell line GES-1. These results indicated downregulated MKRN2 expression in GC; thus, we further analyzed the role and possible mechanism of MKRN2 in GC.

### MKRN2 overexpression inhibits GC cell proliferation *in vitro*

**Figure 2:**
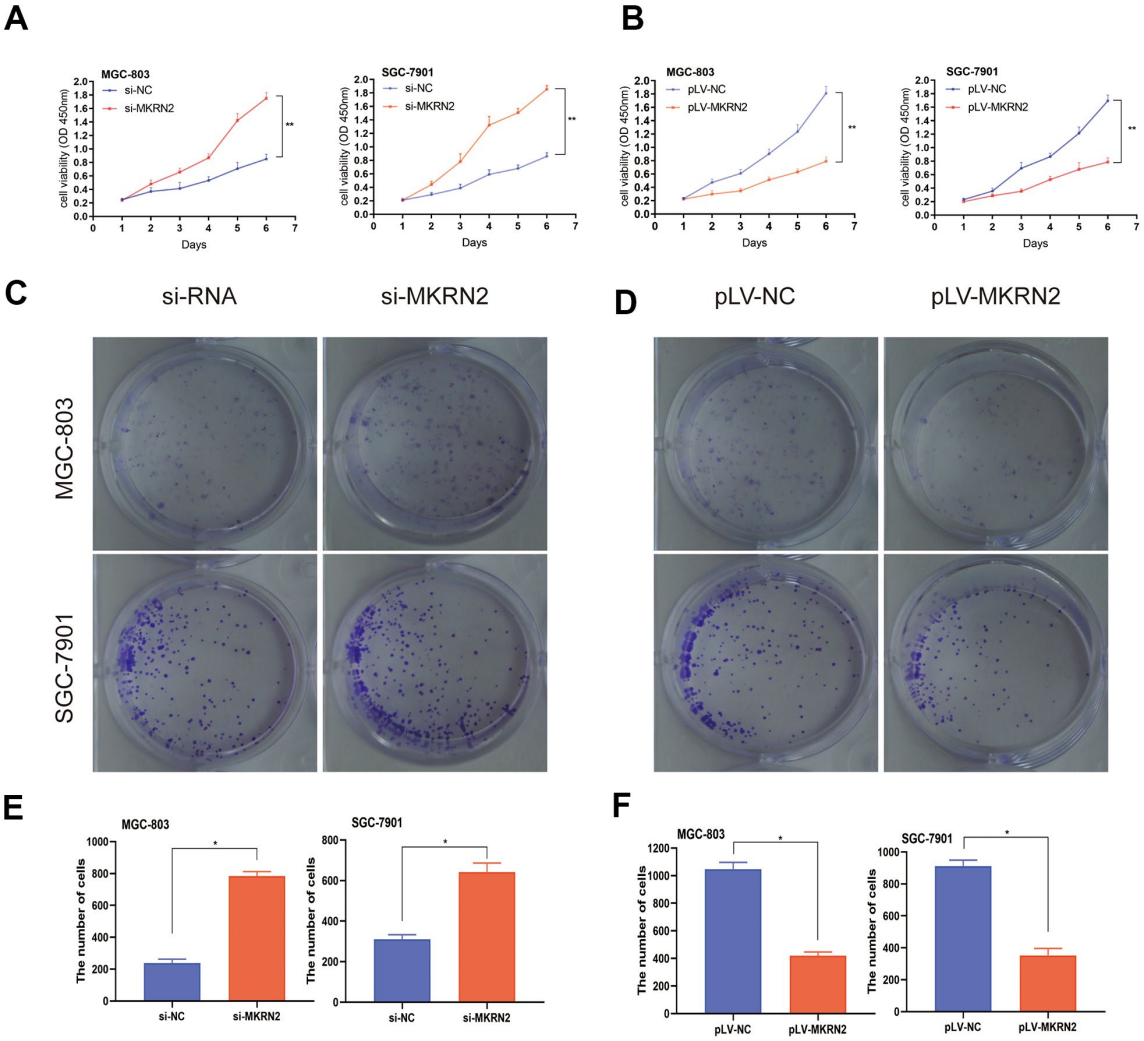
**Effect of MKRN2 modulation on gastric cancer cells *in vitro*.** SGC-7901 and MGC-803 cells were transfected with lentiviral vector containing MKRN2 (pLV-MKRN2) or negative control vector (pLV-NC). Cell proliferation was determined by CCK8 assay and colony formation assay in the SGC-7901 and MGC-803 cells transfected with MKRN2 mimics. Representative photos from the colony formation assay in the SGC-7901 and MGC-803 cells transfected with pLV-MKRN2, pLV-NC. (**A**, **C**, **E**, *: P<0.05). An opposite outcome was found when SGC-7901 and MGC-803 cells were transfected with si-MKRN2 or si-NC (**B**, **D**, **F**,*: P<0.05).

To verify the role of MKRN2 in GC cells *in vitro*, we further established two overexpressed GC cell lines using MGC-803 and SGC-7901, as these cells showed relatively increased expression levels of MKRN2. SGC-7901 and MGC-803 cell lines with MKRN2 overexpression were established by lentiviral transfection (pLV-MKRN2), and MKRN2 mimics as a negative control. The results showed that tumor cell proliferation (Figure 2B) and colony formation (Figure 2D, 2F) were significantly inhibited in the MKRN2-overexpressed group in comparison with that in the control group.

### MKRN2 knockdown promotes GC cell proliferation *in vitro*

We used siRNA to silence MKRN2 gene expression in SGC-7901 and MGC-803 cells and evaluated cell proliferation and colony formation. We observed that si-MKRN2 significantly promoted cell proliferation (Figure 2A) and colony formation (Figure 2C, 2E) compared to the control group (si-NC infected cells).

### MKRN2 overexpression inhibits tumor proliferation *in vivo*

**Figure 3:**
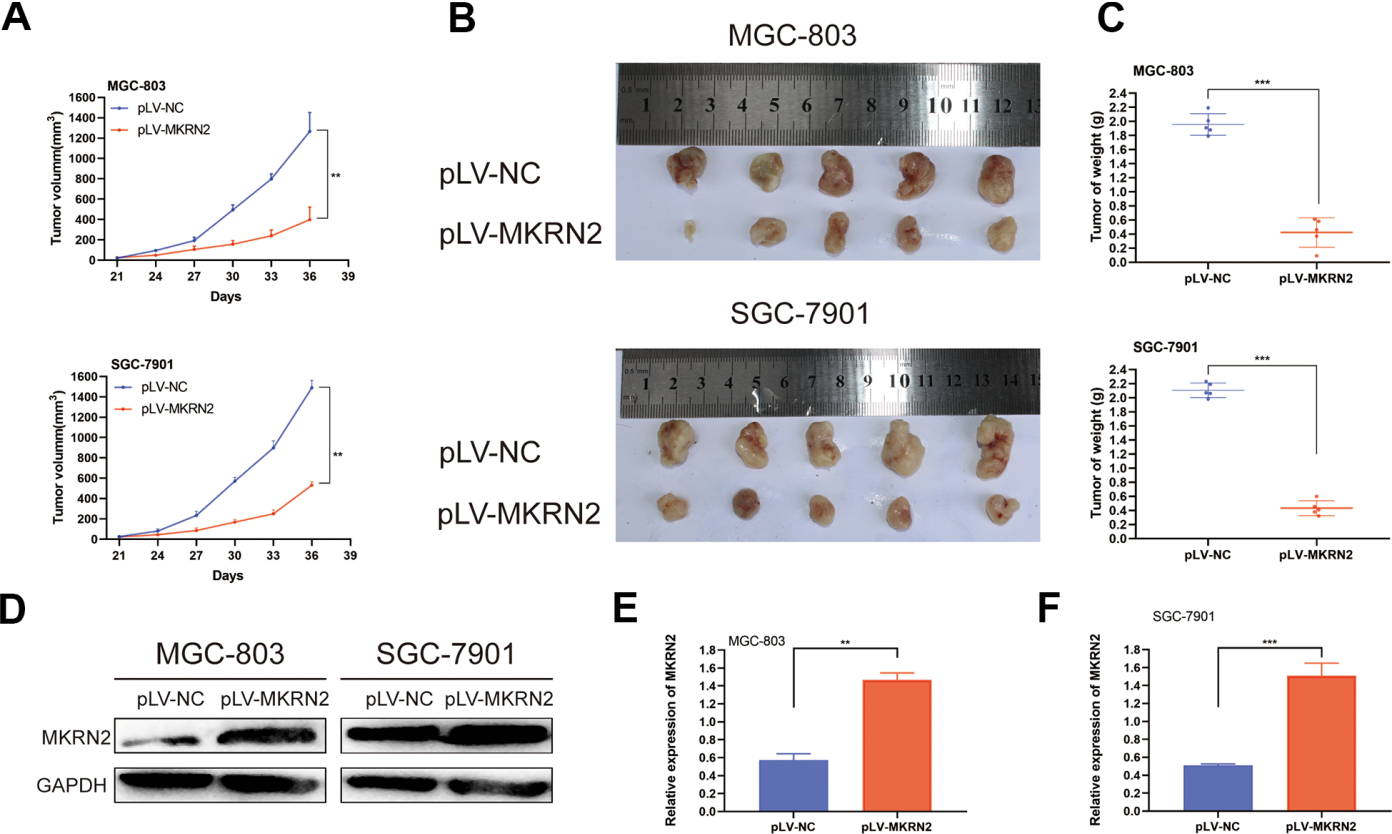
**Overexpression of MKRN2 inhibits xenograft tumor growth *in vivo*.** Xenograft tumors from GC cells with MKRN2 overexpression (MGC-803 and SGC-7901) grew much slower than the tumors from the control cells (transfected with pLV-NC). (**A**) Upon harvest, the xenograft tumors from the MKRN2-overexpressing cells were much smaller in size and weight than the tumors from the control cells (transfected with pLV-NC). (**B**, **C**) Western blot assay showed that the MKRN2 expression of xenograft tumor in experimental group was higher than control group in mice. (**D**–**F**) *: P<0.05, **: P<0.01.

To determine whether an increase of MKRN2 expression suppresses the oncogenesis and growth of GC, we used nude mice bearing a subcutaneous xenograft tumor. Figure 3 shows that tumors derived from mice with pLV-MKRN2-transfected SGC-7901 and MGC-803 cells had significantly slower growth than those from the control group with pLV-NC transfected cells. (Figure 3A, 3B) After sacrifice, we found that the average weight of the xenograft tumors derived from mice with pLV-MKRN2 transfected cells was significantly lower than that of the tumors from the control group (Figure 3C). Hence, we infer that MKRN2 upregulation inhibits the proliferation of GC cells *in vivo*.

### MKRN2 suppresses cell proliferation by inhibiting ERK

**Figure 4:**
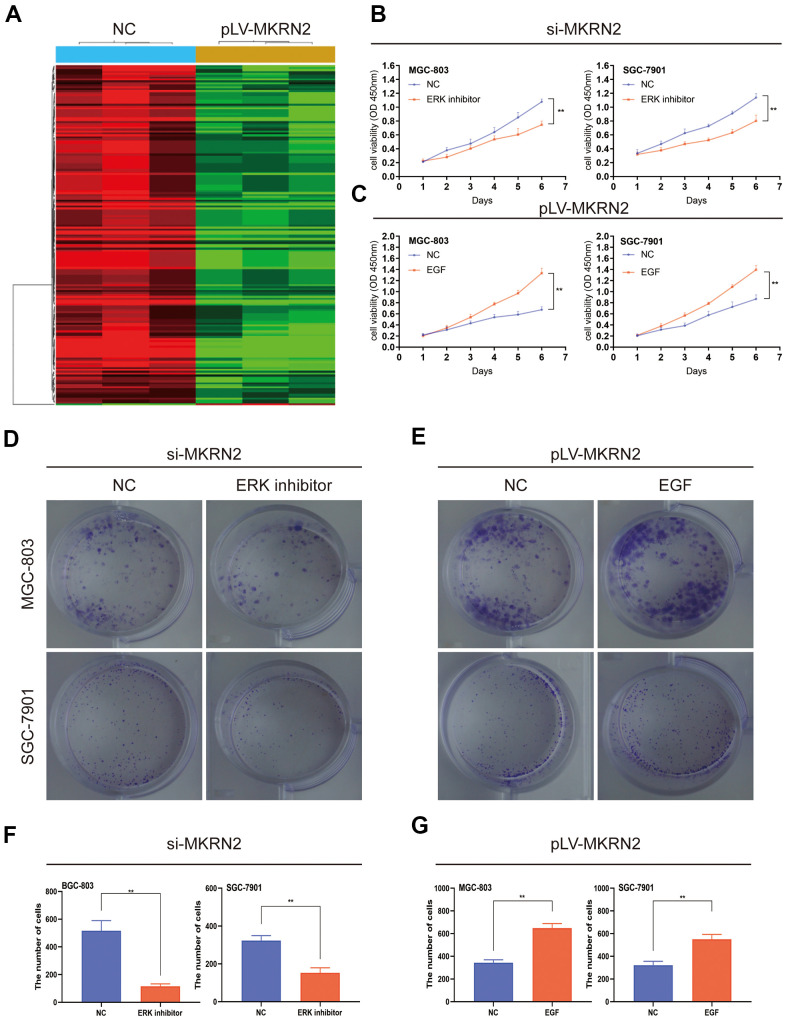
**MKRN2 negatively regulates cell proliferation by inhibiting ERK.** The result of RNA-seq analysis showed MKRN2 affects the ERK signaling pathway (**A**). When ERK activator in SGC-7901 and MGC-803 cells in which MKRN2 was overexpressed, it promoted cell proliferation (**C**, **E**, **G**). However, when used an ERK inhibitor in SGC-7901 and MGC-803 cells in which MKRN2 was inhibited, inhibition of cell proliferation was observed (**B**, **D**, **F**).

To investigate the molecular mechanisms involved in regulating GC by MKRN2, we compared the mRNA expression profiles between pLV-MKRN2 and NC (SGC-7901) by RNA-seq analysis. We found that MKRN2 inhibits the ERK signaling pathway, which plays a vital role in regulating tumor proliferation [12, 13] (Figure 4A).

**Figure 5:**
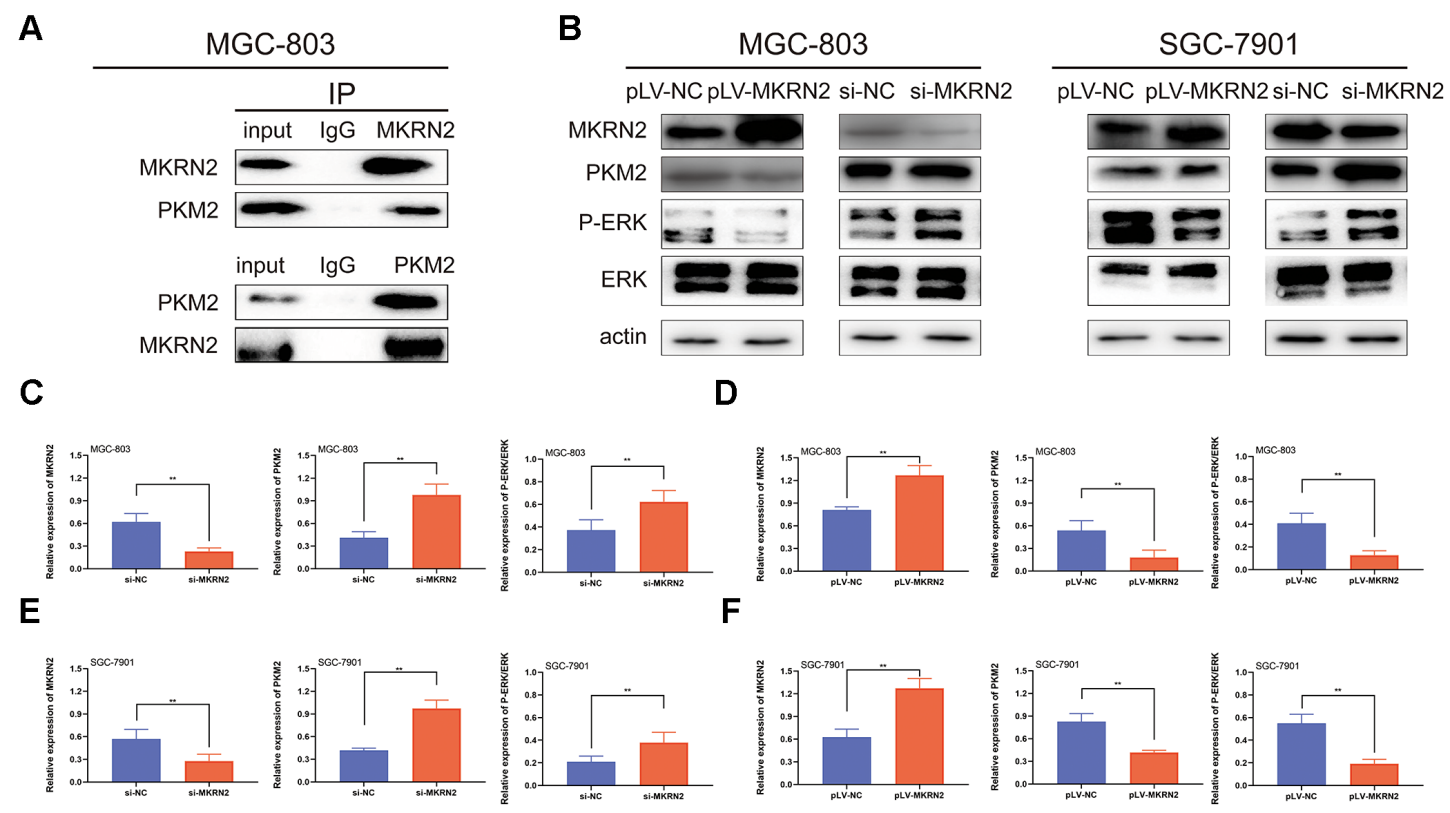
**MKRN2 inhibits PKM2 by promoting its degradation.** Using Co-immunoprecipitation experiments with gastric cells, we showed that MKRN2 was able to coimmunoprecipitate endogenous PKM2 (**A**). MKRN2 decreased ERK phosphorylation by promoting ubiquitination-mediated degradation of PKM2 (**B**). In plv-MKRN2 transfected MGC-803 cells, level of PKM2 and ERK phosphorylation significantly decreased (**D**, **F**). Knockdown of MKRN2 increased the level of PKM2 and ERK phosphorylation (**C**, **E**).

ERK1/2 and P-ERK1/2 protein levels were analyzed using western blot as we altered MKRN2 expression. Our results showed that MKRN2 expression was negatively correlated with P-ERK1/2 levels (Figure 5B).

To further study the ERK1/2 signaling pathway, we used an ERK activator in SGC-7901 and MGC-803 cells in which MKRN2 was overexpressed. We observed that the ERK activator promoted cell proliferation (Figure 4C) and colony formation (Figure 4E, 4G). However, when we used an ERK inhibitor in the two GC cell lines in which MKRN2 was knockdown, inhibition of cell proliferation (Figure 4B) and colony formation (Figure 4D, 4F) was observed.

These findings suggest that MKRN2 inhibits GC cell proliferation by inhibiting ERK.

### MKRN2 inhibits ERK by degrading PKM2

Previous reports shows that various proteins were involved in the regulation of ERK, such as EGFR, PKM2, and MEK [14–16]. To define the regulatory mechanisms of ERK by MKRN2, we assessed proteins associated with ERK activity by Co-immunoprecipitation (Co-IP) and immunoblotting. We found that MKRN2 bound to p65, which meant that MKRN2 inhibited ERK by degrading PKM2 (Figure 5A).

To prove this hypothesis, we further analyzed the expression of PKM2 in SGC-7901 and MGC-803 cells in which MKRN2 was overexpressed or knockdown via western blotting. The results showed that PKM2 expression was inhibited when MKRN2 overexpressed; in contrast, PKM2 expression was promoted (Figure 5B) when MKRN2 was knockdown.

**Figure 6:**
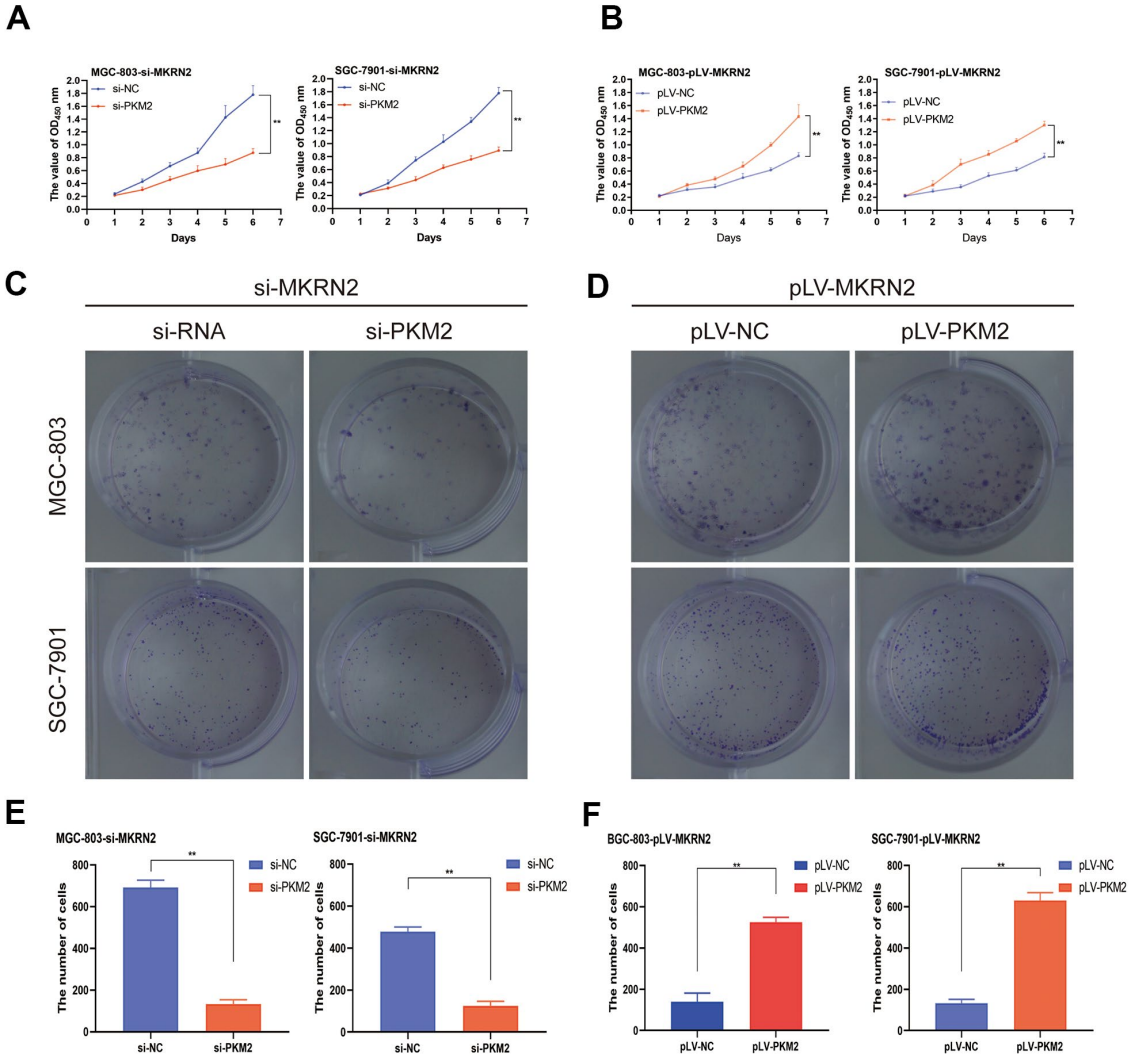
**MKRN2 regulates ERK by degrading PKM2.** When recombinant pcDNA3.1-PKM2 was transiently transfected into SGC-7901 and MGC-803 cells transfected with pLV-MKRN2, it promoted cell proliferation (**B**, **D, F**). However, when siRNA of PKM2 was transiently transfected into SGC-7901 and MGC-803 cells transfected with siRNA-MKRN2, the results were opposite (**A**, **C, E**).

To evaluate the effects of PKM2, recombinant pcDNA3.1-PKM2 was transiently transfected into SGC-7901 and MGC-803 cells transfected with pLV-MKRN2. The recombinant PKM2 promoted cell proliferation (Figure 6B, 6D). However, when siRNA of PKM2 was transiently transfected into SGC-7901 and MGC-803 cells transfected with siRNA-MKRN2, the opposite results were obtained (Figure 6A, 6C).

## DISCUSSION

The development of GC often involves ubiquitination [17, 18]. We found that MKRN2 is poorly expressed in GC by The Cancer Genome Atlas (TCGA) public database. However, the function of MKRN2 in GC remains unknown. Therefore, in the present study, we aimed to reveal the role of MKRN2 in the development of GC.

Mounting evidence suggests that MKRN2 plays an important role in tumor growth and development [19–21]. In our study, we found that MKRN2 is expressed at lower levels in GC tissues than in paracancerous tissues. Clinical correlation analysis showed that the expression level of MKRN2 is related to the size and the degree differentiation of tumor. Furthermore, GC patients with high expression of MKRN2 had better survival rates. This supports the importance of MKRN2 as a marker for preventing poor prognosis.

To investigate the role of MKRN2 in GC, we conducted a series of *in vitro* and *in vivo* experiments. Overexpression of MKRN2 significantly inhibited the proliferation of GC cells when compared with control cells. Likewise, the *in vivo* xenograft model showed that the overexpression of MKRN2 inhibited the growth of GC cells.

To further investigate the molecular mechanisms, RNA-seq and western blot were used to analyze the differential expression of *ERK* and *PKM2* genes in MKRN2 overexpressed GC cells compared to control cells. We found that MKRN2 inhibited the ERK pathway. The rescue experiment further proved that MKRN2 inhibited cell proliferation by inhibiting ERK expression.

Co-IP and western blot analyses showed that MKRN2 could mediate the ubiquitination of PKM2, which promotes cancer proliferation [22]. The evaluation of the effects of PKM2 in GC cells showed that PKM2 plays an important role in the tumor proliferation and P-ERK expression.

In conclusion, the present study revealed that MKRN2 inhibits GC cell proliferation by degrading PKM2. These findings can be useful in diagnosing GC at an early stage, and the knockdown of MKRN2 could potentially lead to the development of better treatment strategies. Further study with a larger patient sample size is necessary in the future.

## MATERIALS AND METHODS

### Cell lines and culture

All cell lines were purchased from the Cell Bank of Chinese Academy of Sciences (Shanghai, China) and were cultured in DMEM (Gibco, NY, USA) supplemented with 10% fetal bovine serum (FBS; Gibco, NY, USA) and with 100 units of penicillin and 100 μg streptomycin per ml in a humidified atmosphere at 37° C with 5% CO_2_.

### Patient samples

A GC tissue microarray, which contained GC tissues (n = 94) and their adjacent normal tissues (n = 86), was purchased from Shanghai Outdo Biotech Company (HStmA180Su19). This study was approved by the Ethics Committee of Chongqing High-tech Zone People’s Hospital.

### Plasmids and adenoviral vectors

Recombinant lentivirus containing MKRN2 and Pyruvate kinase muscle isoform 2 (PKM2) overexpression plasmid and an empty vector were obtained from Shanghai Kaiji Biotech (Shanghai, China).

### RNA interference (RNAi)

Lentiviral siRNAs against the same target genes (MKRN2 and PKM2) and corresponding negative controls were designed, synthesized, and verified by Shanghai Kaiji Biotech (Shanghai, China). The sequence of MKRN2 small interfering RNA (siRNA) was CAGATCACTTGCAGGTATT dTdT, and the sequence of the PKM2 small interfering RNA (siRNA) was “CATCTACCACTTGCAATTA”.

### Cell proliferation and colony formation assay

Proliferation was investigated using the Cell Counting Kit-8 (CCK-8, Dojindo, Japan) assay and colony formation assay. After infection, cells were seeded into 96-well plates (3,000 cells per well) and were incubated at 37° C. Next, CCK-8 solution (10 μl per well) was added, and the absorbance was measured at 450 nm using a microplate reader (Thermo Fisher Scientific, MA, USA). In the colony formation assay, single-cell suspensions were plated (800 cells per dish) with DMEM containing 10% FBS and cultivated. After three weeks, cells were stained with 0.1% crystal violet, and colonies were counted under the microscope.

### Xenograft model

Female BALB/c nude mice (Five-weeks old on average) were obtained from SLAC Laboratory Animal Center (Shanghai, China). Transfected MGC-803 and SGC-7901 cells (1×10^7^ cells per injection) were subcutaneously injected into the flanks of nude mice. All procedures were conducted in accordance with the guidelines of the U.K. Animals (Scientific Procedures) Act, 1986. We calculated the tumor size every 2 days. At the end of the study, the mice were sacrificed and their tumors were removed, photographed, measured, frozen, and stored at -80° C until further analysis.

Tumor size (V) was calculated using the following formula:


$$V=\frac{\text{L}\times {\text{W}}^{2}}{2}$$

L denotes the largest diameter of tumor, whereas W is the shortest diameter. Xenograft experiments were performed in Chongqing High-tech Zone People’s Hospital, Chongqing, China. The experiments were approved by the Ethics Committee of Chongqing High-tech Zone People’s Hospital.

### Western blot assay

Samples were lysed in RIPA buffer (Beyotime Biotechnology, Shanghai, China) for 20 min at 4° C; then, we measured the protein concentrations using a BCA kit (Thermo Fisher Scientific, MA, USA). Next, protein samples were separated on 10% sodium-dodecyl sulfate polyacrylamide gel electrophoresis (40 μg/lane) and were transferred to polyvinylidene difluoride (PVDF) membranes (Sangon Biotech, Shanghai, China). Next, we blocked membranes in PBST with 5% skim milk and incubated the samples at 4° C with rabbit anti-human antibody (anti-MKRN2,1:1000, anti-PKM2,1:1000, anti-ERK,1:2000, anti-actin 1: 3000, anti-p-ERK,1:2000, All antibodies were purchased from Abcam. USA). Finally, horseradish peroxidase (HRP)-conjugated antibody was used as the secondary antibody, and the samples were incubated for 2h.

### RNA sequencing

Three biological replicates samples per group were prepared for the RNA-Seq studies. Total RNA was isolated with TRIzol. The integrity was confirmed with an Agilent 2100 Bioanalyzer. mRNA-Seq libraries were prepared using a TruSeq RNA sample preparation kit version 2 (Illumina). Transcriptome RNA sequencing was performed using the Illumina HiSeq 2000 apparatus.

### Immunohistochemistry

5 μm thick paraffin-embedded tumor tissues were pretreated with 4% formaldehyde. Paraffin-embedded tissue sections were prepared followed by dewaxed, rehydrated, antigen repaired, and endogenous peroxidase activity blocked as well serum blocking. The sections were incubated at 4° C overnight with the primary antibody anti-MKRN2 (1:100), then incubated at 37° C for 1h with the secondary antibody horseradish peroxidase (HRP)-conjugated antibodies (DACO, Kyoto, Japan). The results were investigated by Diaminobenzidine (DAB) Substrate Kit (Vector Laboratories, Inc., Burlingame, CA).

### Co-IP

1 ml lysis buffer contained resuspended cells was used for *co*-immunoprecipitation (*Co*-*IP*) assay. MKRN2 and PKM2 were immunoprecipitated by incubation with 1 μg of respective specified antibodies, followed by western blot with antibodies against MKRN2 (ab228852, Abcam, USA) and PKM2 (ab137852, Abcam, USA). IgG (1 μg) was used as control.

### Statistical analysis

All quantitative data were presented as mean ± standard deviation (S.D.), and qualitative data as a percentage. The difference between groups was compared using unpaired Student’s t-test and Pearson’s chi-square test. Survival data were calculated by the Kaplan-Meier method, and the difference was compared by the log-rank test. All tests were two-sided, and *P* < 0.05 was considered statistically significant.

### Availability of data and materials

Data in this study were available on request, if necessary, please contact the corresponding author.
